# Nearfield control over magnetic light-matter interactions

**DOI:** 10.1038/s41377-025-01807-z

**Published:** 2025-03-19

**Authors:** Benoît Reynier, Eric Charron, Obren Markovic, Bruno Gallas, Alban Ferrier, Sébastien Bidault, Mathieu Mivelle

**Affiliations:** 1 Sorbonne Université, Centre National de la Recherche Scientifique, Institut des NanoSciences de Paris, 75005 Paris, France; 2 Chimie ParisTech, Paris Sciences & Lettres University, Centre National de la Recherche Scientifique, Institut de Recherche de Chimie Paris, 75005 Paris, France; 3 Faculté des Sciences et Ingénierie, Sorbonne Université, UFR 933, Paris, 75005 France; 4 Institut Langevin, ESPCI Paris, Université Paris Sciences et Lettres, Centre National de la Recherche Scientifique, 75005 Paris, France

**Keywords:** Nanophotonics and plasmonics, Single photons and quantum effects, Sub-wavelength optics, Magneto-optics

## Abstract

Light-matter interactions are frequently perceived as predominantly influenced by the electric field, with the magnetic component of light often overlooked. Nonetheless, the magnetic field plays a pivotal role in various optical processes, including chiral light-matter interactions, photon-avalanching, and forbidden photochemistry, underscoring the significance of manipulating magnetic processes in optical phenomena. Here, we explore the ability to control the magnetic light and matter interactions at the nanoscale. In particular, we demonstrate experimentally, using a plasmonic nanostructure, the transfer of energy from the magnetic nearfield to a nanoparticle, thanks to the subwavelength magnetic confinement allowed by our nano-antenna. This control is made possible by the particular design of our plasmonic nanostructure, which has been optimized to spatially decouple the electric and magnetic components of localized plasmonic fields. Furthermore, by studying the spontaneous emission from the Lanthanide-ions doped nanoparticle, we observe that the measured field distributions are not spatially correlated with the experimentally estimated electric and magnetic local densities of states of this antenna, in contradiction with what would be expected from reciprocity. We demonstrate that this counter-intuitive observation is, in fact, the result of the different optical paths followed by the excitation and emission of the ions, which forbids a direct application of the reciprocity theorem.

## Abstract (web summary)

Coupling a europium-doped nanopar9cle to a plasmonic wave shows that both electric and magne9c fields can interact with ma=er.

## Graphical abstract

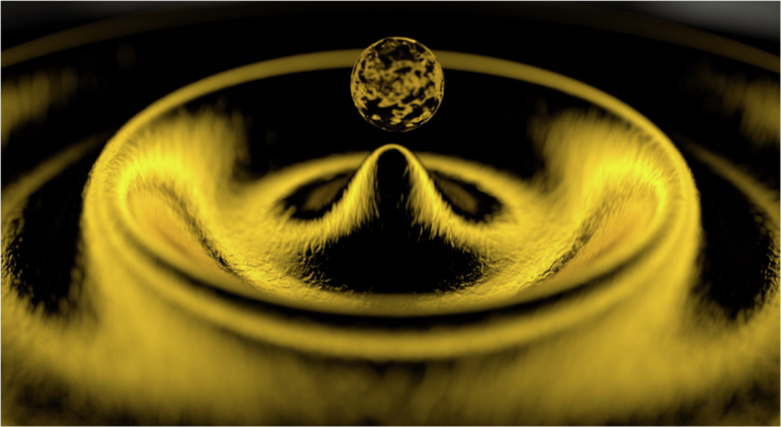

## Introduction

Controlling light-matter interactions at the nanoscale has brought about transformative advancements across various scientific domains. Applications span from high sensitivity in diagnostic platforms for biochemistry^1,2^ to precise nanoparticle-mediated medical therapies^3,4^, increased catalytic efficiency in chemistry^5,6^, and the exploration of exotic light-matter interaction processes in optical physics^7–11^. Despite the substantial progress achieved this far, the focus has been set on manipulating the electric field of light, with the magnetic component often being neglected. Nevertheless, the magnetic field of light assumes a critical role in numerous optical processes, including chiral light-matter interactions^12^, ultrasensitive detection^13^, enhancement of Raman optical activity^14^, photon-avalanching^15^, or forbidden photochemistry^16^. Hence, the manipulation of magnetic processes becomes crucial. In fact, since the early days of nanophotonics, numerous scientific studies have demonstrated the ability to detect and manipulate the magnetic component of light in the near field of photonic nanostructures^17–19^. However, only recent investigations have successfully demonstrated the control over specific interactions involving magnetic light and matter, in particular spontaneous emission^20–41^ and stimulated excitation^41,42^ mediated by magnetic transition dipoles in Lanthanide ions. While the control and enhancement of magnetic luminescence was successfully investigated at scales both larger (through the use of metallic layers as mirrors)^20,21,23,25,30,41^ and smaller than the wavelength of light (thanks to dielectric^22,29,32,34–36,38–40^ and plasmonic nanostructures^26–28,33^) by locally tuning the magnetic density of states, the manipulation of magnetic stimulated excitation was limited to diffraction-limited dimensions, using either a focused azimuthally polarized laser beam^42^ or stationary waves^41^. These pioneering studies have demonstrated that by spatially separating the magnetic field from the optical electric field, it becomes possible to selectively excite nanoparticles doped with trivalent europium ions using either component of the optical field. This selective excitation is achieved by tuning the spectral overlap to match a magnetic or electric dipolar transition. However, just as manipulating the interactions between the electric field of light and matter at the nanoscale has led to significant technological and fundamental advances in photonics, controlling the magnetic component of these interactions at the nanoscale—and thus in the near field of photonic nanostructures— will provide new degrees of freedom to optimize phenomena where the magnetic field is directly involved. In this study, we demonstrate the nearfield control over both stimulated excitation and spontaneous emission in ${{Eu}}^{3+}$ ions thanks to a plasmonic nano-antenna.

Here, we designed a plasmonic nano-antenna with the aim of confining and enhancing the magnetic field at subwavelength scales (See Supplementary Information). Thanks to the properties of light in the near-field, this magnetic hotspot is spatially isolated from its electric counterpart, providing a magnetic nanosource of light with minimal electric field. This antenna is placed at the apex of a near-field scanning optical microscope (NSOM), enabling the magnetic hot spot to be deterministically positioned close to a Lanthanide ion-doped nanoparticle. Through this deterministic coupling between the nano-antenna providing a nanosource of magnetic light and the nanoparticle, we demonstrate the optical excitation of the latter at subwavelength scales by transferring optical energy from the magnetic field to the Lanthanide ions under consideration. This interaction also enables us to independently map the nanoscale distribution of electric and magnetic components of the localized plasmonic fields generated by the nano-antenna, confirming the subwavelength nature of the magnetic field confinement. Also, by studying the spontaneous emission from the doped nanoparticle, we observe that the field distributions are not spatially correlated with the electric and magnetic local densities of states of this antenna, which seemingly contradicts reciprocity^43–46^. We demonstrate that this counter-intuitive observation is in fact the result of the different optical paths followed by the excitation and emission of the nanoparticle, which forbids a direct application of the reciprocity theorem in our experimental configuration.

## Results

### Plasmonic magnetic field excitation of solid-state emitters

**Fig. 1:**
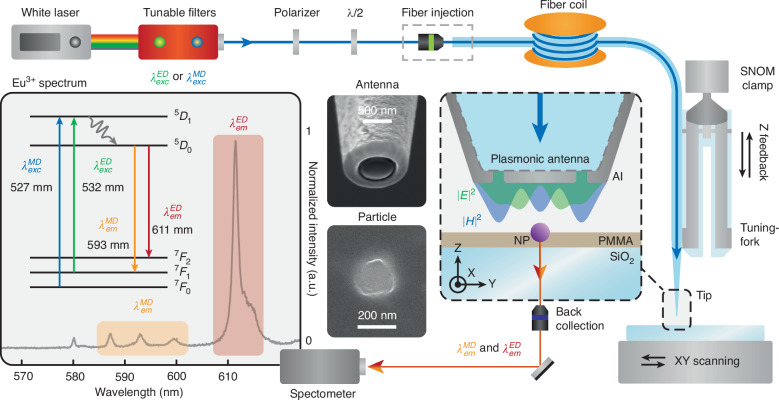
Illustration of the experimental configuration. An aluminum nanodisk serves as a plasmonic nano-antenna and is fabricated at the end of an NSOM fibered tip. This tip is glued to a tuning fork, and via a feedback loop mechanism, the position of the antenna can be deterministically controlled within a few nanometers from the sample, ensuring precise manipulation in all three spatial dimensions. The plasmonic nano-antenna is excited by a supercontinuum laser, filtered using a series of interference filters to isolate a specific wavelength range with a 2 nm bandwidth. The laser beam, rendered linearly polarized, is injected into the optical fiber supporting the tip and antenna, resulting in the optical excitation of the latter. The localized plasmonic fields generated by the antenna are used to excite ***Y***_**2**_***O***_**3**_**:** ***Eu***^**3+**^ nanoparticles, which are deposited on a glass substrate. SEM images of an antenna and of a nanoparticle are shown. Luminescence emitted by the nanoparticle is collected using an immersion objective (×100, NA = 1.3) from the substrate side and measured with a spectrometer. The inset provides the emission spectrum of europium ions in the ***Y***_**2**_***O***_**3**_ matrix, along with the partial band diagram of these emitters. It is important to note that, at the wavelengths under consideration, electric and magnetic dipole transitions dominate the optical response of europium ions, with higher-order processes, such as electric quadrupole transitions, being negligible^51^

The plasmonic nano-antenna used in this study comprises an aluminum nanodisk measuring 50 nm in thickness and 550 nm in diameter, as depicted in Fig. 1. Fabricated at the end of a pulled optical fiber tip, the nano-antenna serves as a local probe for an NSOM when affixed to a tuning fork. This integration offers two crucial advantages. Firstly, it facilitates direct excitation of the nano-antenna through the optical fiber (Fig. 1) by injecting the laser beam directly into the fiber core, allowing its propagation to the tip and to the nanodisk. Moreover, the NSOM's three-dimensional nanometric manipulation of the tip enables precise control of the position of the nano-antenna relative to the particle. In our case, the sample consists of yttrium oxide $({{\boldsymbol{Y}}}_{{\boldsymbol{2}}}{{\boldsymbol{O}}}_{{\boldsymbol{3}}})$ nanoparticles with an approximate diameter of 150 nm, doped with trivalent europium $({{\boldsymbol{Eu}}}^{{\boldsymbol{3}}{\boldsymbol{+}}})$ ions (Fig. 1 and Supplementary Information). These ions in the ${{\boldsymbol{C}}}_{{\boldsymbol{2}}}$ site, of particular interest for this investigation, exhibit purely electric (ED) or magnetic (MD) dipolar transitions for both excitation and emission, as illustrated in the partial band diagram in Fig. 1^47^. These transitions are of the same order of magnitude because, in trivalent lanthanides, electronic transitions within the 4f band are forbidden for electric dipole transitions. They occur only under the influence of the crystal field, which induces “forced” electric dipole transitions^48^. This study leverages these specific properties of europium ions to explore the coupling between magnetic or electric fields and matter at the nanoscale.

**Fig. 2:**
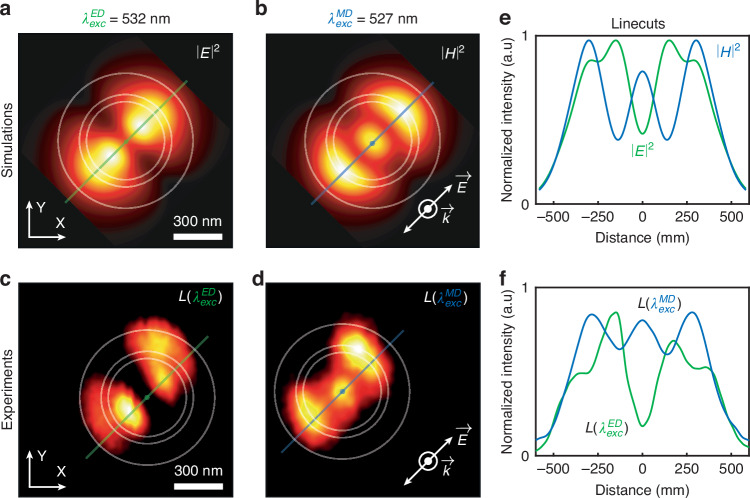
Near-field excitation of the doped nanoparticle by localized plasmonic fields. Theoretical representations of the integrated electric field intensity distribution (**a**, at ${{\boldsymbol{\lambda }}}_{{\boldsymbol{exc}}}^{{\boldsymbol{ED}}}=532{\,\rm{nm}}$) and of the integrated magnetic field intensity distribution (**b**, at ${{\boldsymbol{\lambda }}}_{{\boldsymbol{exc}}}^{{\boldsymbol{MD}}}=527.5{\,\rm{nm}}$) in an XY plane beneath the aluminum nanodisk at a distance Z of 100 nm. The linear polarization of the excitation is as indicated in the inset of b. Normalized luminescence intensities collected while scanning the plasmonic nano-antenna excited at the wavelength of the ED transition (**c,** ${{\boldsymbol{\lambda }}}_{{\boldsymbol{exc}}}^{{\boldsymbol{ED}}}=532{\,\rm{nm}}$) and the MD transition (**d,** ${{\boldsymbol{\lambda }}}_{{\boldsymbol{exc}}}^{{\boldsymbol{MD}}}=527.5{\,\rm{nm}}$). The linear polarization used is shown in the inset of **d**. White circles are guides for the eyes showing the position of the nanoantenna, the gap and the border of the coated tip, the experimental images have a total size of 1.5 μm × 1.5 μm and consist of 50 × 50 pixels. Line cuts are presented for the theoretical field intensities **e** and the experimental luminescence signals **f** obtained from the distributions in **a, b** and **c, d**, respectively. Green lines correspond to line cuts at the ED wavelength, while blue lines correspond to the magnetic counterpart

The dimensions of the nanostructure are meticulously chosen to ensure that under excitation at wavelengths ${{\boldsymbol{\lambda }}}_{{\boldsymbol{exc}}}^{{\boldsymbol{MD}}}=527.5{\,\rm{nm}}$ (MD: ${{\bf{7}}\atop}{\bf{F}}_{{\bf{0}}}{{\to }}{{\bf{5}}\atop}{\bf{D}}_{{\bf{1}}}$) and ${{\boldsymbol{\lambda }}}_{{\boldsymbol{exc}}}^{{\boldsymbol{ED}}}=532{\,\rm{nm}}$ (ED: ${{\bf{7}}\atop}{\bf{F}}_{{\bf{1}}}{{\to}}{{\bf{5}}\atop}{\bf{D}}_{{\bf{1}}}$), the magnetic and electric fields are spatially decoupled in the localized plasmon of the antenna. Additionally, the plasmonic nano-antenna is designed to confine the magnetic field in its core, as illustrated in Fig. 2b. Experimentally, the selection of the excitation wavelength, targeting either the MD or ED transition of ${{\boldsymbol{Eu}}}^{{\boldsymbol{3}}{\boldsymbol{+}}}$ ions, is achieved by finely filtering a supercontinuum laser source. The nanopositioning and feedback capabilities of the NSOM allow the plasmonic nano-antenna to be scanned within a few nanometers and in the plane of the doped nanoparticle, facilitating its near-field excitation by the localized plasmonic fields in the vicinity of the antenna. Since the luminescence signal is theoretically proportional to the targeted exciting field intensity (see Supplementary Information for theoretical details), it is possible to infer a two-dimensional image of the spatial distribution of either the magnetic or electric plasmonic field. Furthermore, by comparing the luminescence signal emanating from the MD (^5^D_0_→^7^F_1_ at ${{\boldsymbol{\lambda }}}_{{\boldsymbol{em}}}^{{\boldsymbol{MD}}}=593{\,\rm{nm}}$) and ED (${{\bf{5}}\atop}{\bf{D}}_{{\bf{0}}}{{\to }}{{\bf{7}}\atop}{\bf{F}}_{{\bf{2}}}$ at ${{\boldsymbol{\lambda }}}_{{\boldsymbol{em}}}^{{\boldsymbol{ED}}}=611{\,\rm{nm}}$) transitions of ${{\boldsymbol{Eu}}}^{{\boldsymbol{3}}{\boldsymbol{+}}}$ (spectrum and band diagram in Fig. 1), at each relative nanoparticle-antenna position, one can also get access to the relative local density of magnetic (MLDOS) and electric (ELDOS) optical states (see Supplementary Information for theoretical details).

Figure 2a, b depict the theoretical distributions of electric (at ${{\boldsymbol{\lambda }}}_{{\boldsymbol{exc}}}^{{\boldsymbol{ED}}}=532{\,\rm{nm}}$) and magnetic (at ${{\boldsymbol{\lambda }}}_{{\boldsymbol{exc}}}^{{\boldsymbol{MD}}}=527.5{\,\rm{nm}}$) field intensities within a plane situated inside the nanoparticle beneath the plasmonic antenna excited by a linearly polarized plane wave (inset in Fig. 2b). A noticeable distinction is observed in the spatial profiles of these fields, characteristic of cavity modes. Specifically, the electric field manifests a two-lobes pattern on the outer regions of the plasmonic nanodisk, whereas the magnetic field displays a three-lobes motif, with one centrally positioned within the disk and two outer lobes in the groove separating the disk from the remainder of the fibered tip. In Fig. 2c, d, the normalized experimental luminescence signal collected at ${{\boldsymbol{\lambda }}}_{{\boldsymbol{em}}}^{{\boldsymbol{ED}}}=611{\,\rm{nm}}$ is presented when scanning a ${{\boldsymbol{Eu}}}^{{\boldsymbol{3}}{\boldsymbol{+}}}$ ion-doped nanoparticle in the vicinity of the plasmonic nano-antenna, as outlined in Fig. 1, when exciting the ED and MD transitions at wavelengths ${{\boldsymbol{\lambda }}}_{{\boldsymbol{exc}}}^{{\boldsymbol{ED}}}=532{\,\rm{nm}}$ and ${{\boldsymbol{\lambda }}}_{{\boldsymbol{exc}}}^{{\boldsymbol{MD}}}=527.5{\,\rm{nm}}$, respectively (See the Supplementary Information for additional luminescence imaging results performed with different tips and on various particles).

The comparison between numerical simulations and experimental outcomes reveals a good agreement. Employing the laser source at the wavelength corresponding to the MD transition effectively leads to the excitation of europium ions through the magnetic field of the localized plasmon. Conversely, employing the wavelength associated with the ED transition results in ion excitation via the electric field. This not only validates the capability to selectively excite matter through the electric or magnetic field of a localized plasmon but also underscores the potential for imaging the full distribution of the field components of light at deep subwavelength scales in the proximity of a plasmonic antenna. This is achieved through the scanning capability of the NSOM and the proportionality of the luminescence signal to the field intensities.

Furthermore, Fig. 2e, f present line cuts of the theoretical and experimental distributions, respectively, as depicted in Fig. 2a–d, with green or blue lines corresponding to the electric and magnetic field distributions. The spatial decoupling of the fields is evident, and again a very good agreement is observed between the simulated field distributions and the spatially dependent luminescence signals. Notably, the magnetic field is prominently localized at the center of the antenna within a subwavelength area.

As in the case of far-field studies^41,42^, these experiments demonstrate the targeted coupling of the magnetic field of a localized plasmon to a nanoparticle at deep subwavelength scales. Moreover, the NSOM capability not only enables selective excitation of matter by the magnetic field but also facilitates the full-scale imaging of light at these spatial dimensions.

### Emission study and reciprocity considerations

The partial band diagram presented in Fig. 1 illustrates that ${{\boldsymbol{Eu}}}^{{\boldsymbol{3}}{\boldsymbol{+}}}$ ions exhibit ED and MD transitions during both excitation and emission processes^41^. Notably, since emission transitions originate from the same energy level, they can serve as a means to characterize the relative radiative MLDOS and ELDOS within a photonic environment, as demonstrated in several studies^21,25,33,34^. Consequently, by examining the luminescence emission, particularly the ratios between the emitted photons in each transition for every position of the plasmonic nano-antenna, the distribution of electric and magnetic LDOS in the vicinity of the plasmonic antenna can be inferred with nanoscale precision (see supplementary materials and methods section for detailed theoretical explanations of the LDOS calculations).

**Fig. 3:**
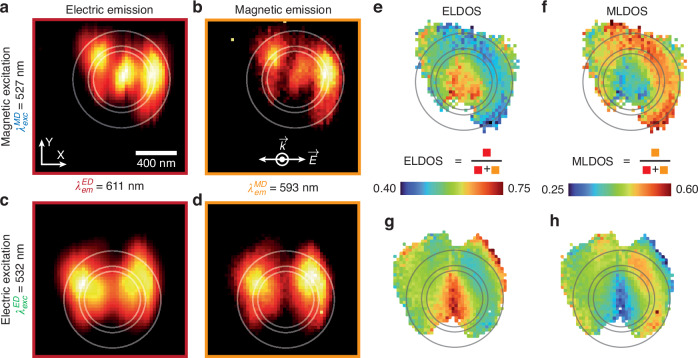
Spatial distributions of electric and magnetic LDOS. Luminescence distributions from a ${{\boldsymbol{Eu}}}^{{\boldsymbol{3}}{\boldsymbol{+}}}$ ion-doped nanoparticle induced by localized plasmon excitation during nanodisk scanning are presented for excitation via (**a**, **b**) the MD transition $({{\bf{7}}\atop}{\bf{F}}_{{\bf{0}}}{{\to }}{{\bf{5}}\atop}{\bf{D}}_{{\bf{1}}})$ at ${{\boldsymbol{\lambda }}}_{{\boldsymbol{exc}}}^{{\boldsymbol{MD}}}=527.5\,{\rm{nm}}$ and (**c**, **d**) the ED transition $({{\bf{7}}\atop}{\bf{F}}_{{\bf{1}}}{{\to }}{{\bf{5}}\atop}{\bf{D}}_{{\bf{1}}})$ at ${{\boldsymbol{\lambda }}}_{{\boldsymbol{exc}}}^{{\boldsymbol{ED}}}=532{\,\rm{nm}}$. These luminescence distributions are further segregated into emission contributions via (**a**, **c**) ED transition (${{\bf{5}}\atop}{\bf{D}}_{{\bf{0}}}{{\to }}{{\bf{7}}\atop}{\bf{F}}_{{\bf{2}}}$, ${{\boldsymbol{\lambda }}}_{{\boldsymbol{em}}}^{{\boldsymbol{ED}}}=611\,{\rm{nm}}$) and (**b**, **d**) MD transition (${{\bf{5}}\atop}{\bf{D}}_{{\bf{0}}}{{\to }}{{\bf{7}}\atop}{\bf{F}}_{{\bf{1}}}$, ${{\boldsymbol{\lambda }}}_{{\boldsymbol{em}}}^{{\boldsymbol{MD}}}=593{\,\rm{nm}}$). Subsequently, the (**e**, **g**) ELDOS and (**f**, **h**) MLDOS are plotted for (**e**, **f**) magnetic and (**g**, **h**) electric excitations, respectively. White and gray circles are guides for the eyes showing the position of the nanoantenna, the gap and the border of the coated tip, the images have a total size of 1.5 μm × 1.5 μm and consist of 50 × 50 pixels

Figure 3 illustrates the ELDOS and MLDOS distributions beneath the antenna. Considering the nearfield control allowed by our experimental approach over both electric and magnetic light-matter interactions, for either stimulated excitation or spontaneous emission, the LDOS can be computed in various manners. Indeed, the plasmonic nano-antenna induces a modulation of the LDOS, allowing the tuning of spontaneous emission from the emitters, independently of the optical excitation. Therefore, the radiative LDOS can be calculated for both electric or magnetic excitations. With this in mind, for each excitation, luminescence signals are distinguished between the contributions of ED (at ${{\boldsymbol{\lambda }}}_{{\boldsymbol{em}}}^{{\boldsymbol{ED}}}=611{\,\rm{nm}}$) and MD (at ${{\boldsymbol{\lambda }}}_{{\boldsymbol{em}}}^{{\boldsymbol{MD}}}=593{\,\rm{nm}}$) transitions. It should be noted here that the radiative LDOS is not considered vectorial because the emission properties of lanthanides originate from transitions between 4f levels defined by spin-orbit coupling. These transitions maintain spherical symmetry in both excitation and emission, resulting in an averaging over all orientations.

In Fig. 3a, b, the luminescence distribution collected respectively at ${{\boldsymbol{\lambda }}}_{{\boldsymbol{em}}}^{{\boldsymbol{ED}}}$ and ${{\boldsymbol{\lambda }}}_{{\boldsymbol{em}}}^{{\boldsymbol{MD}}}$, are shown, using a magnetic excitation at ${{\boldsymbol{\lambda }}}_{{\boldsymbol{exc}}}^{{\boldsymbol{MD}}}$. Similarly, Fig. 3c, d display, for the same emission wavelengths, the collected luminescence distribution with an electric excitation at ${{\boldsymbol{\lambda }}}_{{\boldsymbol{exc}}}^{{\boldsymbol{ED}}}$. Upon initial examination, the two image sets appear identical at a specific excitation wavelength, reflecting the described distributions of electric or magnetic plasmonic fields. However, subtle variations are noticeable, especially at the center of the image when considering either the magnetic or electric emission: for both excitation conditions, magnetic emission appears relatively weaker than electric emission. For a more quantitative analysis, these photoluminescence maps enable the calculation of the relative electric and magnetic radiative LDOS for both electric and magnetic excitations, as shown in Fig. 3e–h. Specifically, Fig. 3e, g provide the ELDOS for magnetic and electric excitations, respectively, whereas Fig. 3f, h present the MLDOS corresponding to these excitation conditions (See the Supplementary Information for additional LDOS imaging results performed with different tips and on various particles).

**Fig. 4:**
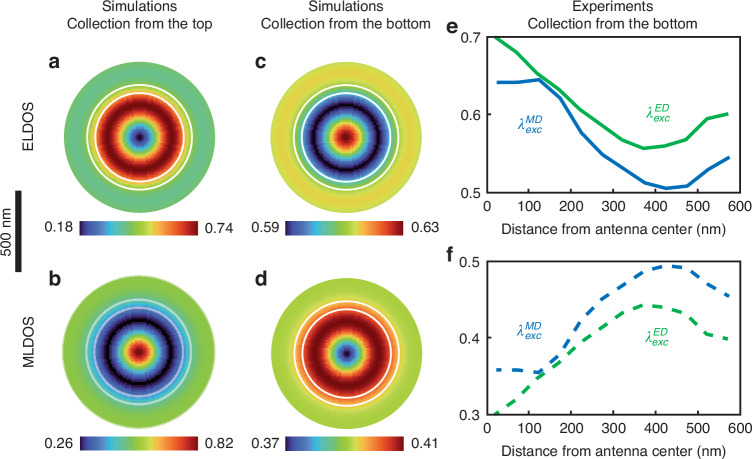
Comparative analysis of the radiative magnetic and electric LDOS for spontaneous emission towards the substrate or in the opposite direction. The distributions of electric (**a**, **c**) and magnetic (**b**, **d**) radiative LDOS are presented for radiation in the direction of the NSOM tip (**a**, **b**) and the substrate (**c**, **d**). Experimental ELDOS (**e**) and MLDOS (**f**) for electric (green lines) and magnetic (blue lines) excitations, starting from the center of the nanodisk to its edge. Experimental curves are obtained by averaging the LDOS provided in Fig. 3e–h in a circular pattern from the center of the nanodisk to increase the signal-to-noise ratio. White circles are guides for the eyes showing the position of the nanoantenna and the gap

Several key observations emerge from this analysis. The estimated LDOS distributions display similar trends whether the doped nanoparticle is excited through an electric or magnetic transition. Notably, the ELDOS tends to be maximum at the center of the antenna, coinciding with a minimum of the MLDOS. Note that the luminescence considered is incoherent, so the MLDOS and ELDOS are independent of the excitation. However, these quantities can only be calculated where photons are emitted; this is why the LDOS maps in Fig. 3e–h only sample a part of the LDOS and present spatial differences compared to the numerical calculations (Fig. 4).

This finding was unexpected since, in Fig. 2, it is the magnetic field, not the electric, which is typically maximum in the center of the antenna. According to the reciprocity theorem, local field enhancements should mirror the distributions of the radiative LDOS^43–46^. This apparent anomaly underscores the interest of mapping independently both excitation and emission processes in the nearfield, for either electric or magnetic transitions, in order to identify and analyze such inconsistencies.

The reason why reciprocity cannot be directly applied to analyze these experimental measurements lies in the distinct optical paths followed during laser excitation and the collection of photoluminescence signals. The reciprocity theorem, which asserts equality when emitter and detector positions are swapped, can be extended to the radiative decay rate of a quantum source and the associated exciting field if the source emission and the excitation wave follow the same optical path, i.e. if the emission and excitation share the same wavevectors with opposite signs^43–46^. In our experimental setup, excitation is performed through the optical fiber, while the luminescence is collected by the immersion objective. Consequently, the optical paths are opposite, explaining the absence of reciprocity in this context.

Numerical simulations confirm this hypothesis. In Fig. 4a comparison is presented between the theoretical electric and magnetic LDOS beneath the plasmonic antenna, and the experimental data. Figure 4a–d depict the distributions of electric (Fig. 4a, c) and magnetic (Fig. 4b, d) radiative LDOS in two different directions—along the tip direction (positive Zs, Fig. 4a, b) and towards the substrate (negative Zs, Fig. 4c, d).

In these simulations, the antenna is modeled as an infinite layer of aluminum, disregarding the tip. However, for a better analogy with the experimental setup, the collection of the luminescence signal in the substrate accounts for the numerical aperture of the microscope objective (Fig. 1). Moreover, the signal collected in the upper part considers only the energy radiated directly above the nanodisk (see materials and methods section for simulation details). As one can see, the spatial distribution of the magnetic and electric LDOS are completely reversed. While the ELDOS is maximum in the center of the antenna for radiation towards the substrate, it is minimal for radiation towards the upper part, and vice versa for the MLDOS. A comparison with the experimental results shown in Figs. 3e–h and 4e, f indicates that the experimental distributions align well with theoretical LDOS radiated towards the substrate. These results shed new light on the limits of the reciprocity theorem, especially when considering the magnetic component of light, which, to our knowledge, has never been discussed in the literature.

## Discussion

Our study leveraged a plasmonic nano-antenna to demonstrate the selective excitation of a solid-state nanoparticle by the magnetic field of a localized plasmon. The nanoscale confinement of the magnetic field by the nano-antenna revealed that this transfer of energy occurs at strongly subwavelength scales. Through precise targeting of excited optical transitions, we achieved nearfield imaging of all electric and magnetic components of the localized plasmonic fields within the nano-antenna. The versatility in selecting the exciting field, coupled with the ability to analyze spontaneous emission via electric and magnetic transitions in the doped particle, allowed the nanoscale imaging of the spatial distributions of electric and magnetic relative LDOS that are manipulated by the antenna at a subwavelength scale.

Furthermore, based on these LDOS distributions, our analysis demonstrated that the reciprocity theorem, applied to the magnetic field of light, could not be applied here due to the different optical paths taken by the optical excitation and the collected luminescence emission. Notably, this study of the reciprocity theorem applied to the magnetic field represents, to our knowledge, the first experimental report on this subject.

The manipulation of the coupling between magnetic light and matter at the nanoscale, particularly through plasmonic nano-antennas, unveils promising prospects across various research domains, such as chiral light-matter interactions^12^, photochemistry^16^, manipulation of magnetic processes^49^, and new schemes in quantum computing^50^ or nonlinear processes^15^, among others.

## Materials and methods

### Plasmonic nanodisk fabrication

The fabrication process for the nanostructured tips involves multiple sequential steps. Initially, an optical fiber is pulled using a P-2000 puller from Sutter to create the fibered tip. Subsequently, a layer of 120 nm aluminum is deposited around the perimeter of the fibered tip, primarily for focused ion beam (FIB) purposes. The tip is then precision-cut by a focused ion beam to achieve a core diameter of approximately 800 nm. Following this, a second layer of aluminum, 50 nm in thickness, is thermally evaporated onto the processed end section of the tip. Lastly, a circular groove is created using FIB to form a nanoantenna with a diameter of 550 nm.

### Nanoparticles synthesis

2% Eu:Y_2_O_3_ nanoparticle of 150 ± 50 nm average diameter were prepared by homogeneous precipitation^38^. In this method, an aqueous nitrate solution of Y(NO_3_)_3_. 6H_2_O (99.9% pure, Alfa Aesar), Eu(NO_3_)_3_. 6H_2_O (99.99% pure, Reacton) was mixed with an aqueous urea solution (CO(NH_2_)_2_, >99% pure, Sigma) in a Teflon reactor. The pH inside the reactor was then slowly increased during a 24 h thermal treatment at 85 °C by the urea decomposition. The metal and urea concentrations were 7.5 mmol L^−1^ and 3 mol L^−1^ respectively. After cooling, a white precipitate of amorphous yttrium hydroxycarbonate (Eu^3+^: Y(OH)CO_3_.n H2O) was collected by centrifugation and washed at least 3 times with water and absolute ethanol. That amorphous powder was then converted to highly crystalline Eu:Y_2_O_3_ nanoparticles by 24 h calcination treatment at 1000 °C (rate of 3 °C min^−1^). The body-centered cubic Y_2_O_3_ structure (Ia-3 space group) of the particles was confirmed by X-ray diffraction with no evidence for other parasitic phases.

### Sample preparation

After cleaning by sonic bath and plasma cleaner, a 110 nm layer of PMMA is deposited by spin-coating (3% weight −4000 rpm) on glass coverslips then annealed at 180° for one minute to evaporate the excess solvent and homogenize the layer. To make the layer hydrophilic, the sample is once again treated with plasma cleaner, reducing the PMMA layer to 80 nm thickness. The doped nanoparticles are then deposited by spin-coating on the sample, and new annealing is performed at 180° for one minute to allow the nanoparticles to embed in the polymer.

### Simulations

The simulations were conducted using the finite difference time domain (FDTD) software Lumerical. The aluminum nano-antenna, positioned at the end of a fibered tip, features a diameter of 550 nm with a metal thickness of 50 nm. This metal structure is separated from the remainder of the tip by a 50 nm gap. The optical index of the glass fiber tip is 1.46, and the permittivity of aluminum employed in the simulation was determined experimentally using ellipsometry. Beneath the tip, a spherical Y_2_O_3_ nanoparticle with a diameter of 150 nm and an optical index of 1.94 is introduced. This nanoparticle is positioned on a glass substrate (index of 1.5) and is partially embedded in a 100 nm layer of PMMA (index of 1.46). The overall size of the simulation window is approximately 2 × 2 × 1.5 µm³, and the finest mesh, defining the most detailed parts, has a resolution of 5 nm.

For investigating electric and magnetic field distributions, a Gaussian beam was introduced into the fiber at 800 nm, directed towards the plasmonic antenna. Additionally, to consider the impact of the nanoparticle size, an integration of electric and magnetic fields within the nanoparticle was performed for each position beneath the nano-antenna. This approach yielded a qualitatively simulated luminescence distribution. The electric and magnetic fields were calculated at ${\lambda }_{{exc}}^{{MD}}$ and ${\lambda }_{{exc}}^{{ED}}$, respectively. The results were normalized by the intensities of electric and magnetic fields of the incoming plane wave.

A simplified model was employed to analyze the ELDOS and MLDOS, as well as to examine the reciprocity theorem. Electric and magnetic dipoles were positioned beneath a 50 nm thick 2D infinite aluminum layer featuring a 550 nm diameter disk with a 50 nm gap to model the plasmonic antenna. The considered electric and magnetic dipoles were emitting at ${\lambda }_{{em}}^{{ED}}$ and ${\lambda }_{{em}}^{{MD}}$, respectively. For each dipole orientation, the radiated power was collected from below, considering the experimental Numerical Aperture (NA), and from above, specifically just on top of the antenna center, at the origin position. To mimic the isotropic orientation of the ${{Eu}}^{3+}$ ions, the results for each dipole oriented along X, Y, and Z were averaged. Similar simulations were conducted without the aluminum layer for the radiated power references (labeled by 0 subscripts). The position of the dipoles is then scanned below the antenna to provide the maps of the ELDOS and MLDOS. Finally, a convolution over the nanoparticle size is performed and provides the ELDOS and MLDOS experienced by the emitters as featured in Fig. 4 of the main text.

### Optical and near-field experimental setup

Excitation of Eu^3+^-doped nanoparticles is performed by a supercontinuum laser (NKT Photonics K90-110-10), filtered by a combination of interference filters (Semrock BrightLine FF01-532/18-25 and Spectrolight FWS-B-F06), in order to reduce the spectral bandwidth to 2 nm while maintaining high laser power. First, the excitation light is finely polarized and injected into the optical fiber. Then, the end of the fiber coil is welded to the optical fiber supporting the nano-antenna. The optical near-field microscope (NT-MDT-Integra) is placed on an inverted microscope (Olympus IX73), and the tip supporting the nano-antenna is glued on a tuning fork vibrating at a frequency of 32kHz. The approach and the feedback loop of the tip in the near field are performed by monitoring the phase of the oscillation of the tuning fork (oscillation below 1 nm). Next, the tip is aligned on the center of an oil immersion objective (Olympus PLN 100× Oil Immersion, NA 1.30), and the particle is scanned under it thanks to a piezoelectric stage (Piezoconcept), allowing a nanometric displacement. Then, the luminescence is collected from below and sent to a spectrometer (Sol Instruments MS5204i) after high-pass filter (Semrock BrightLine FF552-Di01-25x36). The luminescence spectra are then measured with a CCD camera (Andor iDus 401 CCD) for each particle-antenna position, leading to hyperspectral images.

## Supplementary information

Supplementary Information - Nearfield Control over Magnetic Light-Matter Interactions

## Data Availability

The materials, data and code underlying the results presented in this paper are not publicly available at this time but may be obtained from the authors upon reasonable request.
