# Supplementary material for: Nearfield control over magnetic light-matter interactions
Source: Light Sci Appl. 2025 Mar 19;14:127. doi: 10.1038/s41377-025-01807-z (PMC11923067; doi:10.1038/s41377-025-01807-z)
Supplement: Supplementary file 1 — Supplementary Information - Nearfield Control over Magnetic Light-Matter Interactions [file 41377_2025_1807_MOESM1_ESM.pdf]

Supplementary Information

Nanoscale Control over Magnetic Light-Matter Interactions

Benoît Reynier (benoit.reynier@inps.jussieu.fr),[1]
Eric Charron (eric.charron@insp.jussieu.fr),[1]
Obren Markovic (obren.markovic@insp.jussieu.fr),[1]
Bruno Gallas (bruno.gallas@insp.jussieu.fr),[1]
Alban Ferrier (alban.ferrier@chimieparistech.psl.eu),[2,3]
Sébastien Bidault (sebastien.bidault@espci.fr),[4]
Mathieu Mivelle (mathieu.mivelle@sorbonne-universite.fr)[1*]

[1] Sorbonne Université, Centre National de la Recherche Scientifique, Institut des NanoSciences de Paris, 75005 Paris, France
[2] Chimie ParisTech, Paris Sciences & Lettres University, Centre National de la Recherche Scientifique, Institut de Recherche de Chimie Paris, 75005 Paris, France
[3] Faculté des Sciences et Ingénierie, Sorbonne Université, UFR 933, Paris 75005, France
[4] Institut Langevin, ESPCI Paris, Université Paris Sciences et Lettres, Centre National de la Recherche Scientifique, 75005 Paris, France

[*]Corresponding author (mathieu.mivelle@sorbonne-universite.fr, +33 1 44274442)

Eu^{3+}ion-doped Y_2O_3 nanoparticles

The europium ions Eu^{3+} offer the potential for excitation through various transitions. In particular, the transition at $\lambda_{exc}^{MD} = 527.5$ nm and the transition at $\lambda_{exc}^{ED} = 532$ nm have been shown to be mediated by magnetic and electric transition dipoles, respectively[1]. Figures S1a and b display the excitation spectra achieved by scanning the excitation wavelength in increments of 1nm and by collecting the electric and magnetic dipole emission peaks at $\lambda_{em}^{ED} = 611$ nm and $\lambda_{em}^{MD} = 593$ nm.[2,3]

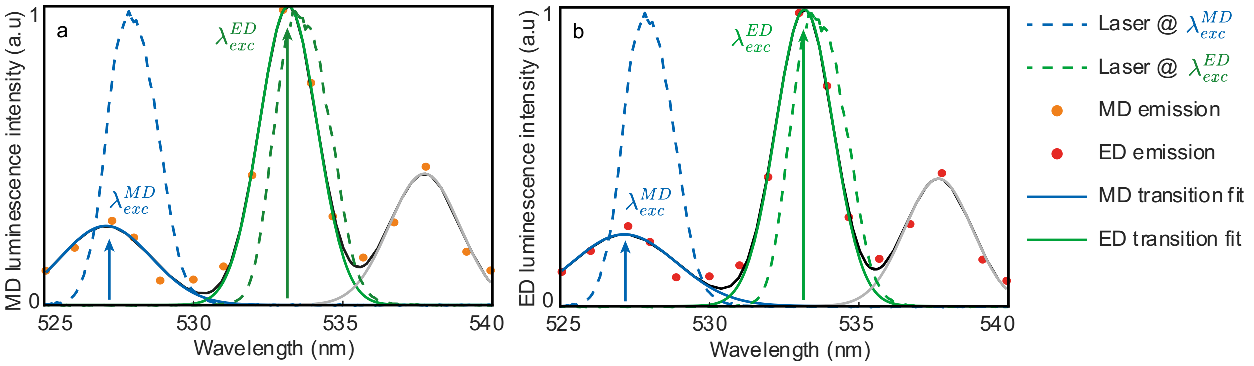

Figure S1. a) Excitation spectrum of Eu^{3+}-doped nanoparticles for a collection at $\lambda_{em}^{MD} = 593$ nm ($^5D_0 \rightarrow {}^7F_1$). b) Excitation spectrum of Eu^{3+}-doped nanoparticles for a collection at $\lambda_{em}^{ED} = 611$ nm ($^5D_0 \rightarrow {}^7F_2$). The laser lines used in the study to excite the electric and magnetic transitions are shown as green and blue dashed lines, respectively. The corresponding electric ($^7F_1 \rightarrow {}^5D_1$) and magnetic ($^7F_0 \rightarrow {}^5D_1$) transitions are shown as green and blue solid line fits. As we can see, the excitation peaks are independent of the emission channel.

Theoretical background: excitation study

The luminescence L at the emission wavelength λ_i for the i transition (i = ED or MD), excited by the field A (with A the electric E or magnetic H field) can be defined as:

$$L(A, \lambda_i) = \sigma(A) \times |A|^2 \times \eta(\lambda_i) \times Q(\lambda_i),$$

where, $\sigma(A)$ is the absorption cross-section, $|A|^2$ is the electric or the magnetic field intensity, $\eta(\lambda_i)$ and $Q(\lambda_i)$ are the collection efficiency and the quantum yield of the transition, respectively.

Since the electric and magnetic excitations occur at different wavelengths ($\lambda_{exc}^{MD} = 527.5$ nm and $\lambda_{exc}^{ED} = 532$ nm), one might think that the plasmonic fields distributions would not be the same, due to a different response of the plasmonic antenna to the incident light. The field intensity distributions are

given in Figure S2 for both incident wavelengths. We do not see any major variations in spatial distributions and in local intensity.

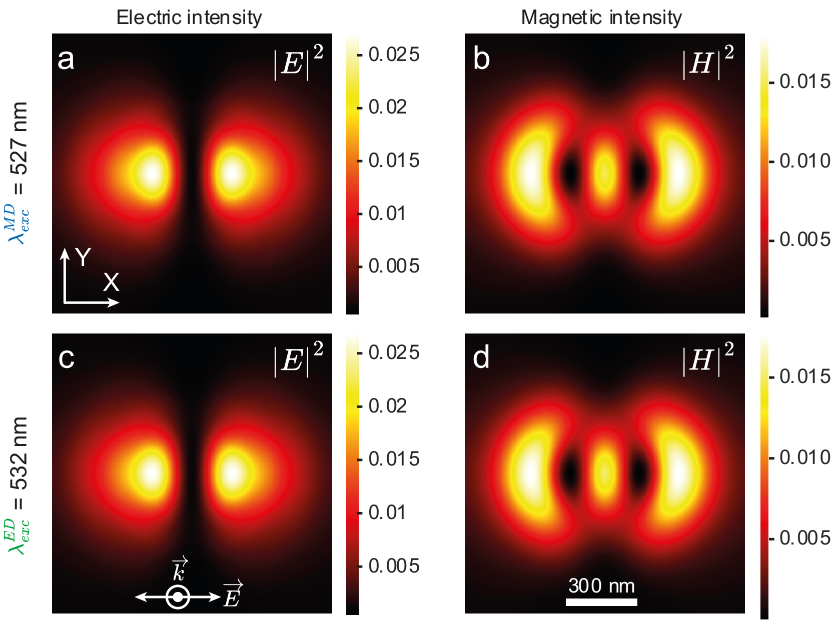

Figure S2. a) Electric and b) magnetic intensity distributions at λ_{exc}^{MD} = 527.5 nm and c) electric and d) magnetic intensity distributions at λ_{exc}^{ED} = 532 nm. All the distributions share the same incident polarization, scalebar and XY observation plane which is 100 nm below the plasmonic antenna. These simulations were performed without considering the luminescent nanoparticle.

Theoretical background: calculation of the LDOS

Introduced by Fermi's golden rule, the local density of states (LDOS) describes how the spontaneous emission of an electric (or a magnetic) dipole quantum emitter is influenced and linked to its surrounding environment. Exploiting the electric ($^5D_0 \rightarrow {}^7F_2$) and magnetic ($^5D_0 \rightarrow {}^7F_1$) dipolar transitions emanating from the same excited level, Eu^{3+} ions serve as ideal candidates for probing the electric and magnetic radiative LDOS. Consequently, one can compute for each spectrum the corresponding electric β^{ED} and magnetic β^{MD} branching ratios using the following expressions:

$$\beta^{ED} = \frac{\Gamma^{ED}}{\Gamma^{ED}+\Gamma^{MD}} = \frac{L^{ED}}{L^{ED}+L^{MD}} = 1 - \beta^{MD},$$

where L^{MD} and $L^{ED}$are the collected luminescence at λ_{em}^{MD} = 593 nm and λ_{em}^{ED} = 611 nm, respectively, with Γ^{MD} and Γ^{ED}, their corresponding radiative decay rates. These branching ratios describe the competition for an excited electron to radiate energy either through an electric or a magnetic dipole

transition. Next, by comparing the calculated branching ratios in the presence of the plasmonic antenna (β_{PA}) to a reference situation without the antenna and the nanoparticle excited from the farfield (β_0), one can directly compute the relative variation of the electric (or magnetic) radiative local density of states, denoted as ELDOS (or MLDOS) using the formula[4]:

$$\tilde{\rho}^{ED} = \frac{\rho_{PA}^{ED}/\rho_0^{ED}}{\rho_{PA}^{ED}/\rho_0^{ED} + \rho_{PA}^{MD}/\rho_0^{MD}} = \frac{\beta_{PA}^{ED}/\beta_0^{ED}}{\beta_{PA}^{ED}/\beta_0^{ED} + \beta_{PA}^{MD}/\beta_0^{MD}} = 1 - \tilde{\rho}^{MD}$$

Here, we can experimentally get access to the influence of the plasmonic antenna on the spontaneous emission of an electric dipole with respect to a magnetic dipole. A calculated ELDOS higher than 0.5 means that the spontaneous emission of the electric dipole is favored with respect to the magnetic dipole. Conversely, an ELDOS lower than 0.5 means that the spontaneous emission of the magnetic dipole is favored with respect to the electric dipole.

The variation of the ELDOS and MLDOS due to the presence of the plasmonic antenna can also be calculated numerically, by knowing that:

$$\frac{\rho_{PA}}{\rho_0} = \frac{\Gamma_{PA}}{\Gamma_0} = \frac{P_{PA}}{P_0},$$

where Γ_{PA} and Γ_0 are the radiative rates experienced for a dipole with the plasmonic antenna and without it, respectively. P_{PA} and P_0 are the corresponding numerically calculated radiated powers (see the Methods section for more details).

Additional luminescence images:

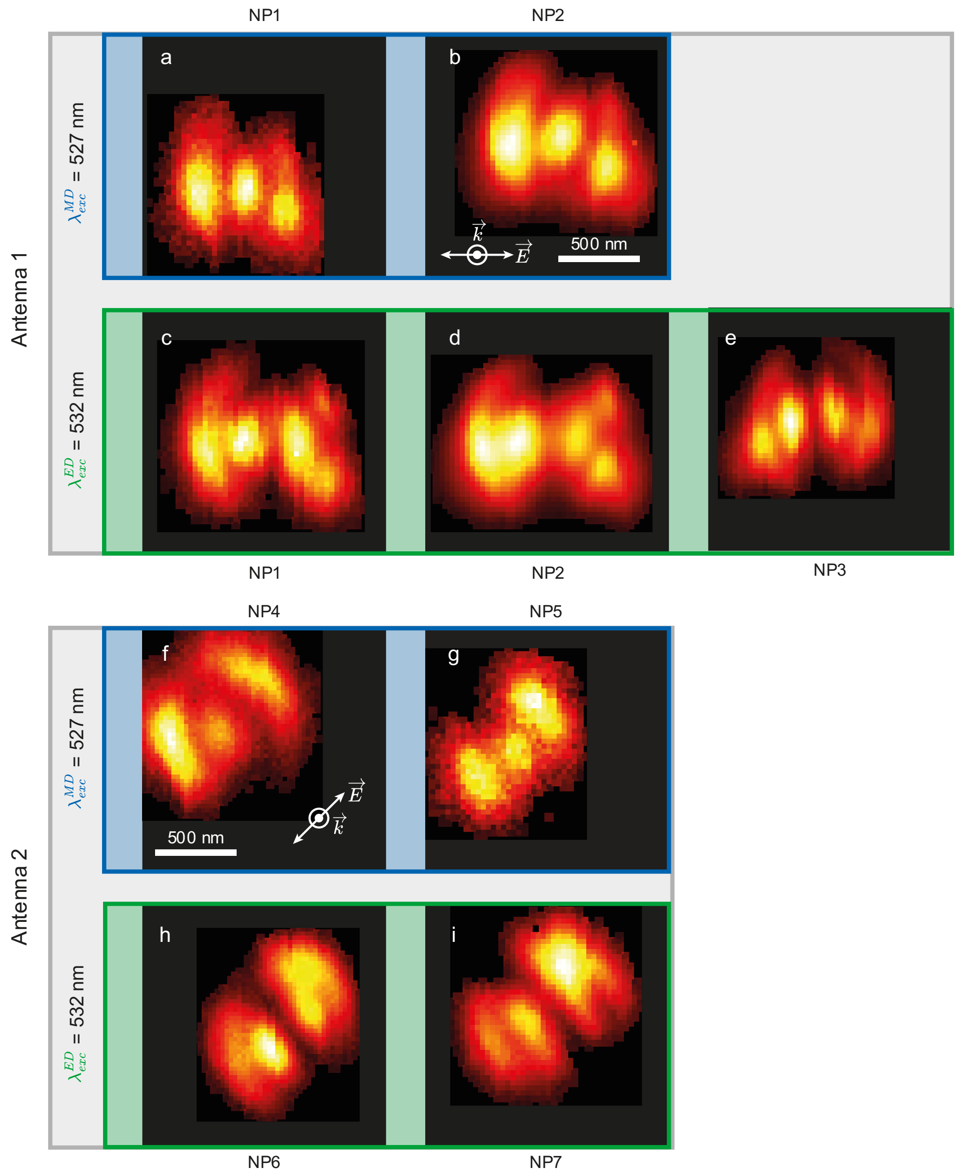

Figure S3. Additional Luminescence Images. Two different scanning probe tips featuring plasmonic antennas with the same dimensions, denoted as Antenna 1 (a-e) and Antenna 2 (f-i), were employed to excite different europium-doped nanoparticles both magnetic (a,b and f,g) and electric (c,d,e and h,i) wavelengths. All luminescence images are normalized and share the same size scale. a) and c) luminescence images have been done with the same nanoparticle (NP1), as well as b) and d) (NP2). Note that g) and h) provide the dataset showed in Figure 1. All measurements provide similar results that are in good agreement with the electric and magnetic fields simulated in the near-field of the antenna. Small discrepancies probably arise from the size and shape of the nanoparticle and from a potential tilt of the fiber impinging on the sample.

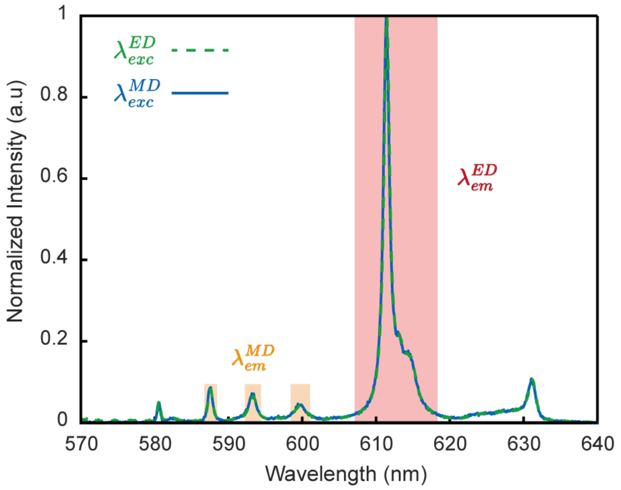

Figure S4. Reference spectrum used for the LDOS calculations. The nanoparticle is excited at $\lambda_{\text{exc}}^{\text{MD}} = 527.5$ nm or at $\lambda_{\text{exc}}^{\text{ED}} = 532$ nm.

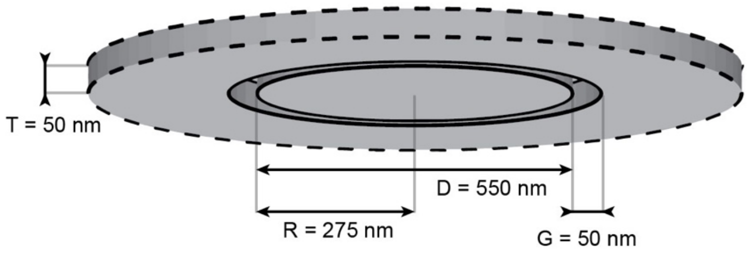

Figure S5. Dimensions of the optimized plasmonic nano-antenna designed to maximize the magnetic field at the center of the nanodisk relative to the electric field at a wavelength of 527.5 nm

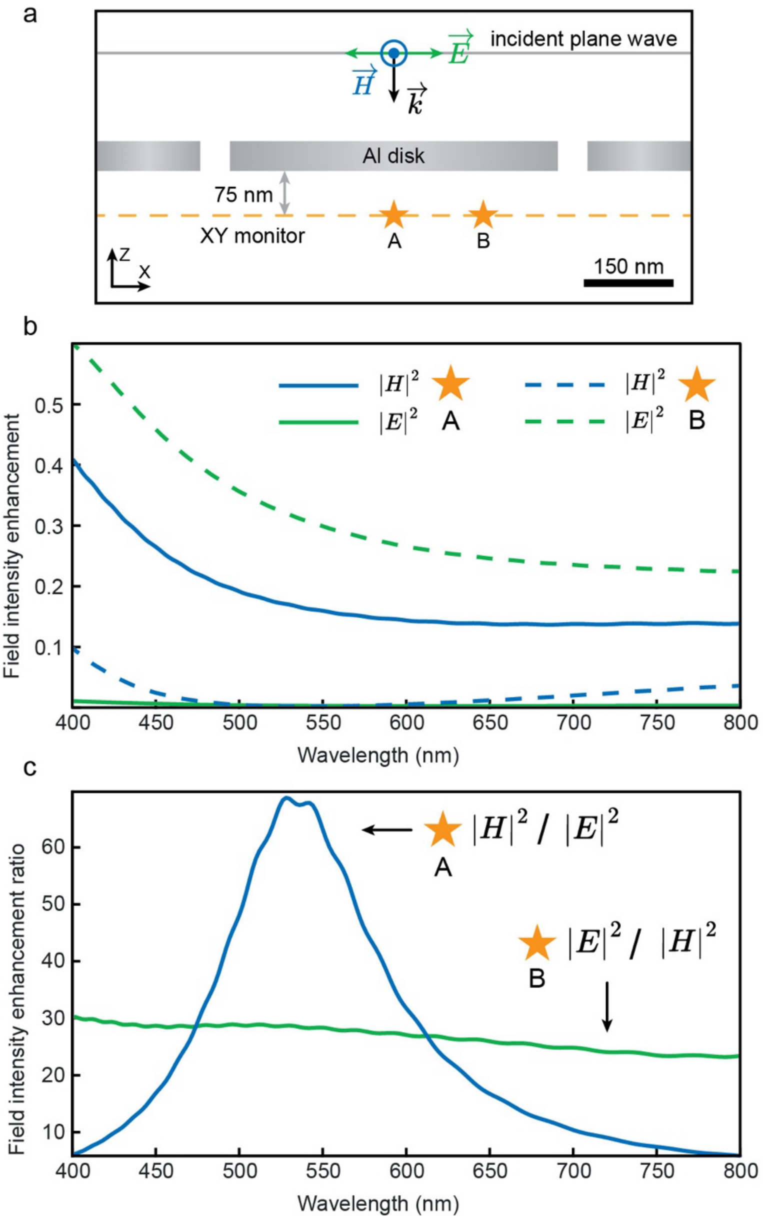

Figure S6. Optimization of the dimensions of the plasmonic nanoantenna. a) Schematic of the simulation parameters: an aluminum nanodisk of 50 nm thickness is excited from the backside, normal to the surface, by a plane wave linearly polarized along X. The electric fields E and magnetic fields H are then analyzed at two different positions, at the center (A) of the antenna and at its edge (B), as symbolized by the yellow stars. Points A and B are placed 75 nm below the disk to account for the thickness of the particle scanned experimentally. b) Spectral responses of the electric and magnetic intensities for the two positions A and B indicated in a). c) Spectral responses of the ratios of magnetic to electric intensities and electric to magnetic intensities, respectively at points A and B, as indicated in A). As can be seen, the ratio of magnetic to electric intensities at the center of the antenna is maximal for the europium excitation transitions (electric at λ_{exc}^{ED} = 532 nm and magnetic at λ_{exc}^{MD} = 527.5 nm).

The plasmonic nanoantenna behaves like a plasmonic cavity with an interference pattern within this cavity—that is, with nodes and antinodes. Due to the symmetry of our plasmonic nanoantenna, at a given wavelength, the positions of the plasmon's nodes and antinodes are generally at the same locations. Specifically, the magnetic field always interacts constructively at the center of the antenna, while the electric field interacts destructively (Figure S7 and S9). This is due to the particular symmetry of our system, where the two slits of the nanoantenna produce two coherent light sources that generate plasmonic electric fields out of phase and in-phase magnetic fields (Figure S8).

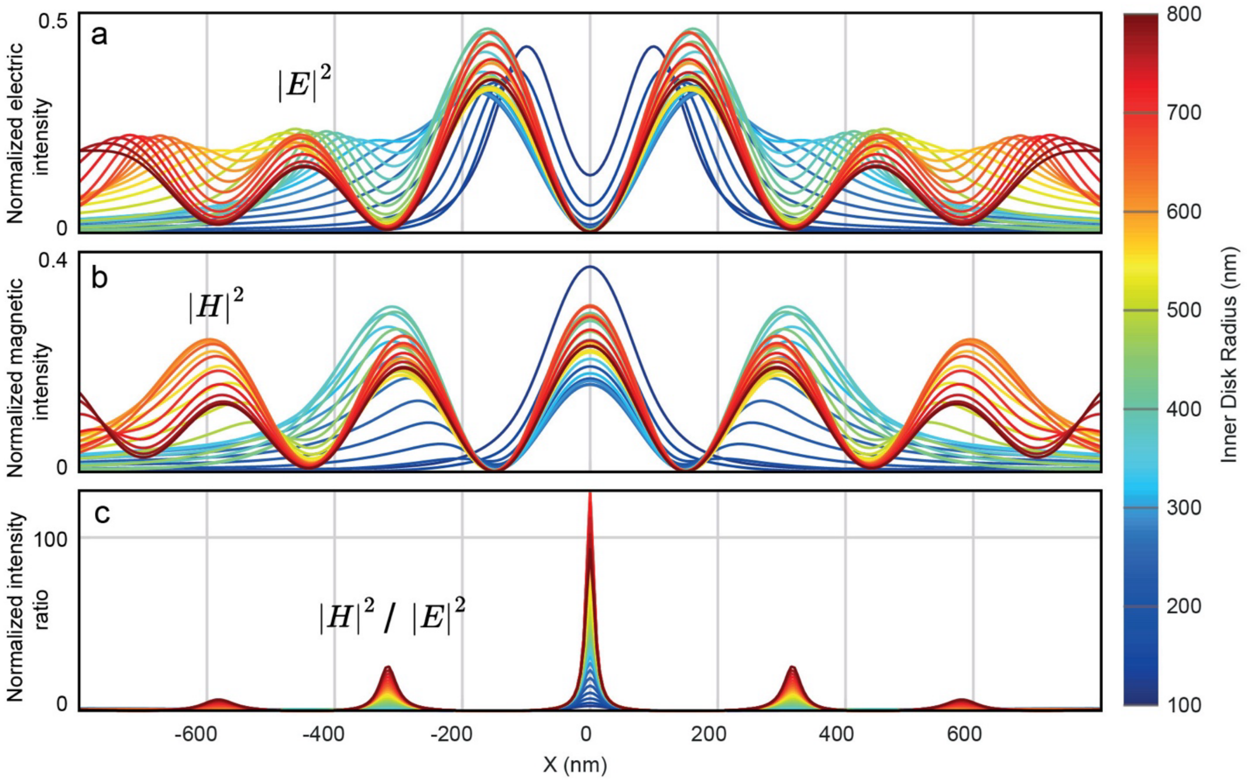

Figure S7. Separation of Electric and Magnetic Fields in the Localized Surface Plasmon. a) Electric and b) magnetic intensities in an XZ plane at 75 nm from the antenna for different radii of the nanodisk. c) Ratio of magnetic to electric intensities from the curves shown in a) and b).

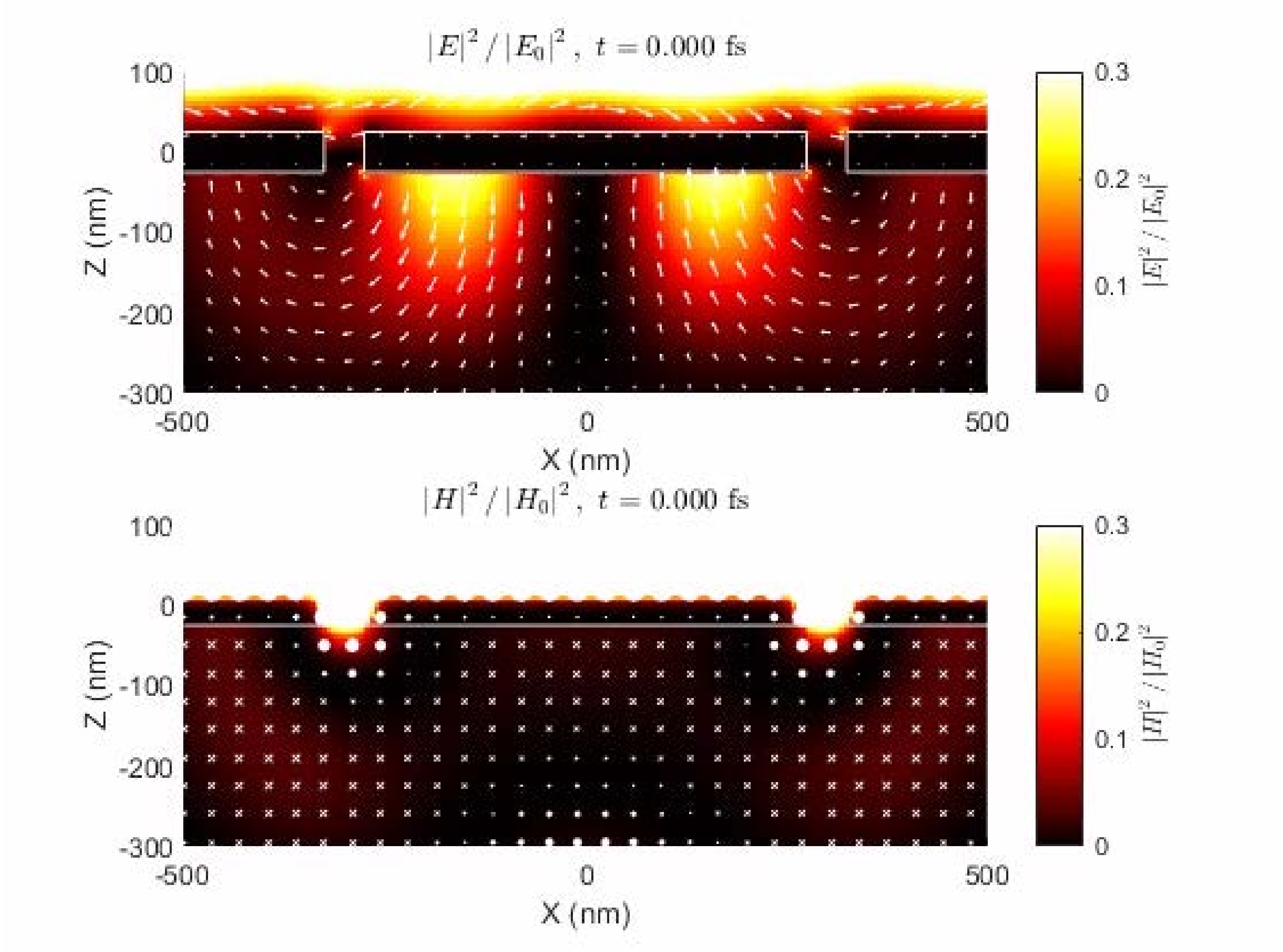

Figure S8. Animation during one optical cycle of the constructive and destructive interferences of the electric (top) and magnetic (bottom) intensities in an XZ plane passing through the plasmonic antenna. The colors represent the intensity; the arrows, dots, and crosses represent the vector direction of the fields.

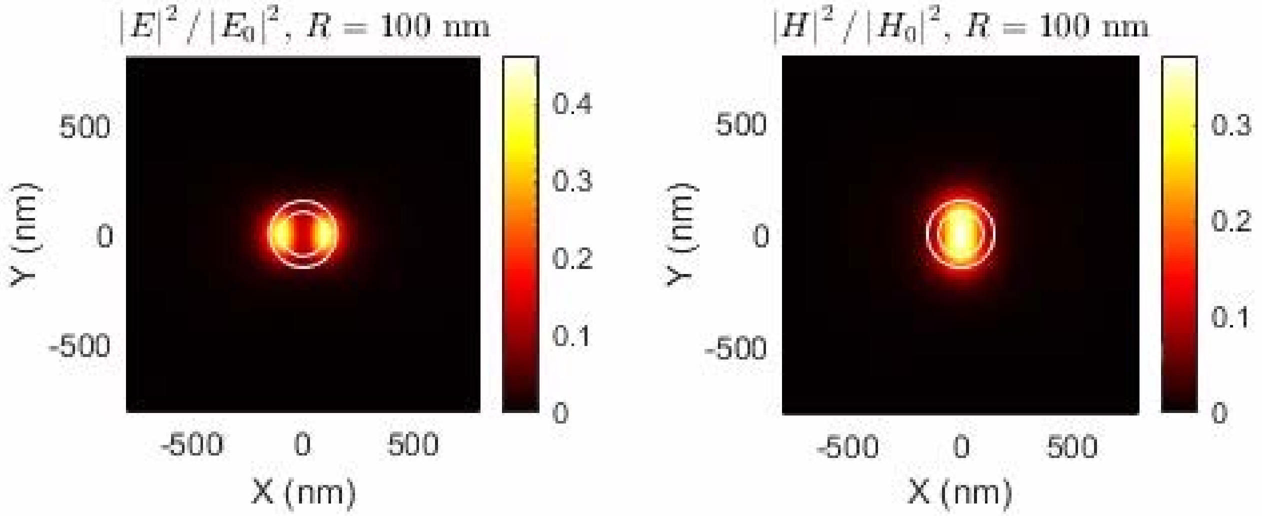

Figure S9. Animation showing the distribution of electric and magnetic field intensities beneath the plasmonic antenna in an XY plane at 75 nm from the antenna, as a function of the nanodisk radius.

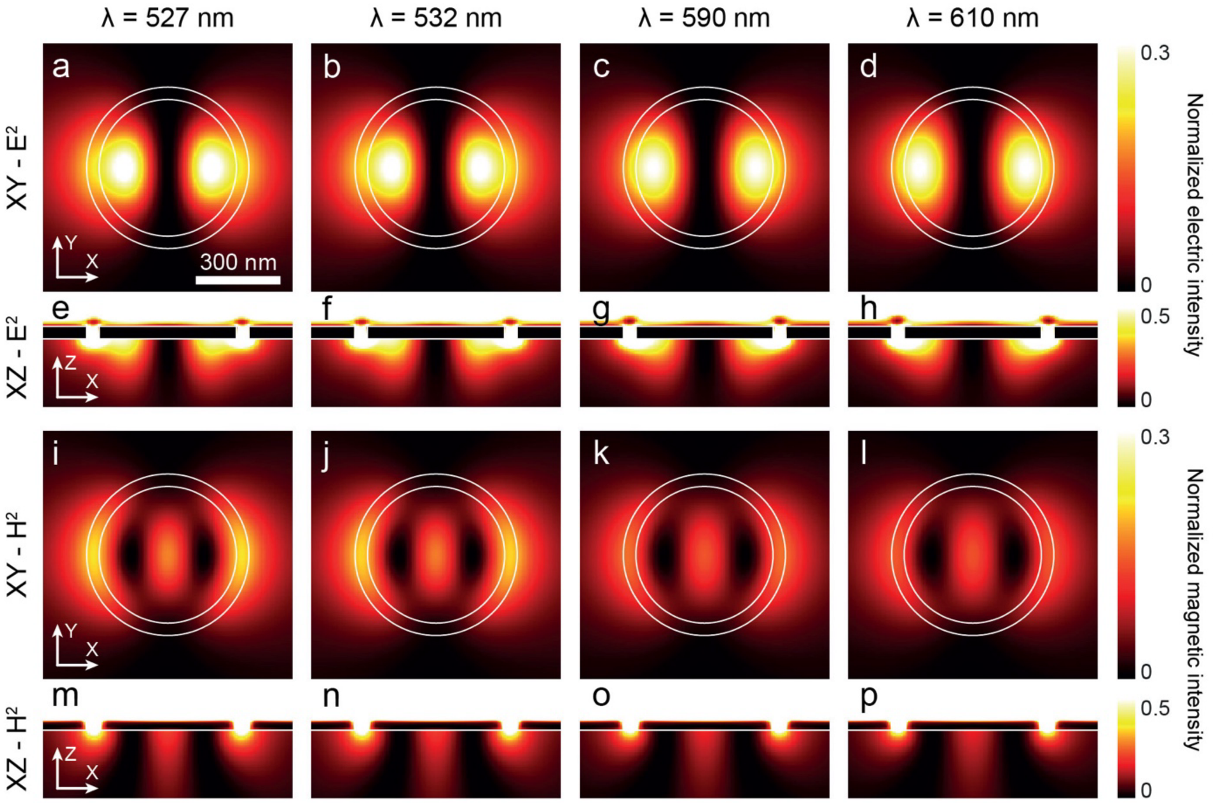

Figure S10. Intensity distributions of a–h) electric and i–p) magnetic fields in an a-d,i-l) XY plane at 100 nm below the antenna, and e-h,m-p) XZ plane in the center of the disk, for excitation by a plane wave linearly polarized along X, at wavelengths of a,e,i,m) $\boldsymbol{\lambda_{exc}^{MD}}$ = 527 nm, b,f,j,n) $\boldsymbol{\lambda_{exc}^{ED}}$ = 532 nm, c,g,k,o) $\boldsymbol{\lambda_{em}^{MD}}$ = 590 nm, et d,h,l,p) $\boldsymbol{\lambda_{em}^{ED}}$ = 610 nm.

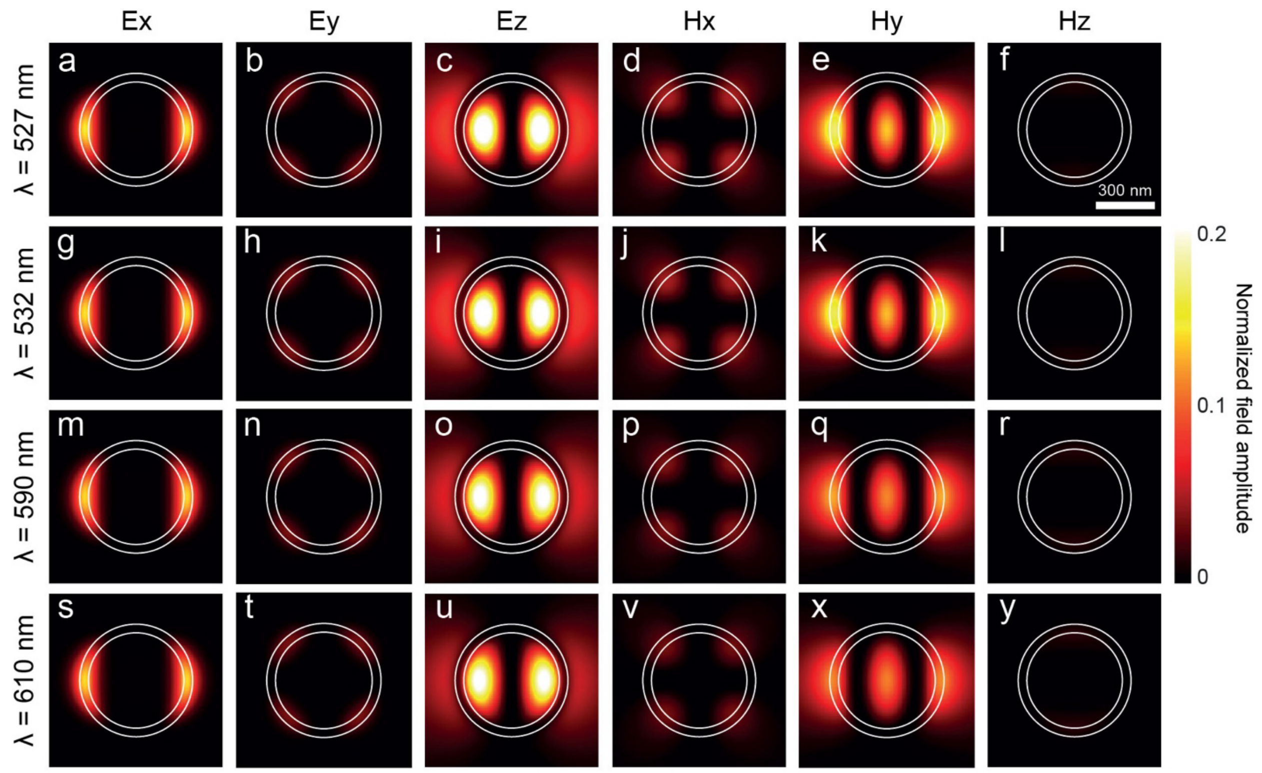

Figure S11. Vectorial distribution of the intensities of (a-b, g-l, m-o, s-u) electric and (d-f, j-l, p-r, v-y) magnetic fields in an XY plane at 100 nm below the antenna for excitation by a plane wave linearly polarized along X, at wavelengths of a-f) $\boldsymbol{\lambda_{exc}^{MD}}$ = 527 nm, g-l) $\boldsymbol{\lambda_{exc}^{ED}}$ = 532 nm, m-r) $\boldsymbol{\lambda_{em}^{MD}}$ = 590 nm, et s-y) $\boldsymbol{\lambda_{em}^{ED}}$ = 610 nm. a,d,g,j,m,p,s,v) represents the X components of the fields, (b,e,h,k,n,q,t,x) the Y components, and (c,f,I,l,o,r,u,y) the Z components.

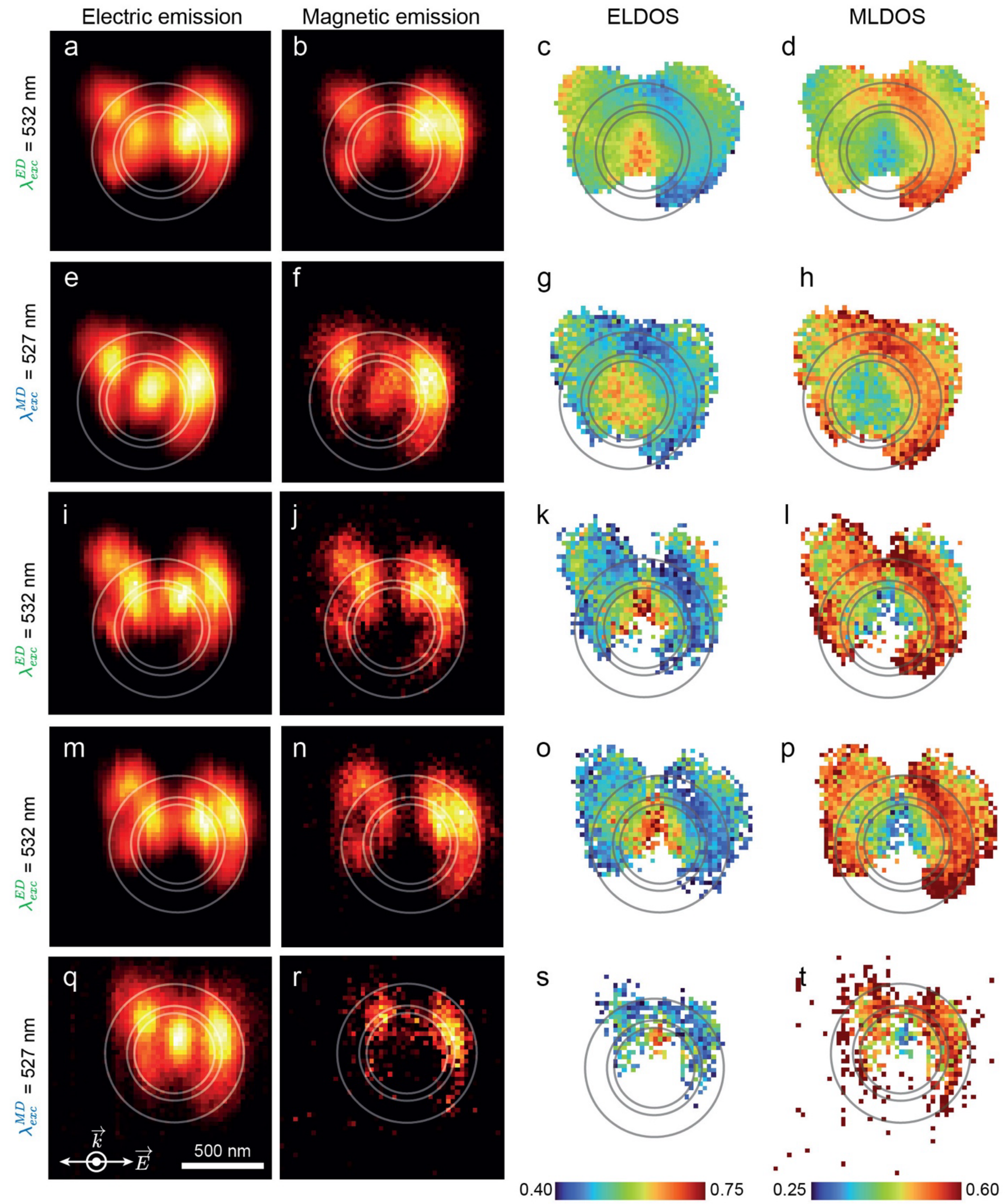

Figure S12. Spatial distributions of electric and magnetic LDOS. Luminescence distributions from a $\boldsymbol{Eu^{3+}}$ ion-doped nanoparticle induced by localized plasmon excitation during nanodisk scanning are presented for excitation via **a-d, i-l, m-p** the ED transition ($\mathbf{^7F_1 \rightarrow {}^5D_1}$) at $\boldsymbol{\lambda_{exc}^{ED}}$ = 532 nm and **e-h, q-t** the MD transition ($\mathbf{^7F_0 \rightarrow {}^5D_1}$) at $\boldsymbol{\lambda_{exc}^{MD}}$ = 527.5 nm. These luminescence distributions are further segregated into emission contributions via **a,e,i,m,q**) ED transition ($\mathbf{^5D_0 \rightarrow {}^7F_2}$, $\boldsymbol{\lambda_{em}^{ED}}$ = 611 nm) and

b,f,j,n,r) MD transition ($^5\mathbf{D_0} \rightarrow {}^7\mathbf{F_1}$, λ_{em}^{MD} = 593 nm). Subsequently, the **c,g,k,o,s**) ELDOS and **d,h,l,p,t**) MLDOS are plotted for **c,d,k,l,o,p** electric and **g,h,s,t** magnetic excitations, respectively. White and gray circles are guides for the eyes showing the position of the nanoantenna, the gap and the border of the coated tip, the images have a total size of 1.5 µm x 1.5 µm and consist of 50 x 50 pixels.

References:

1. Kasperczyk, M. et al. Excitation of magnetic dipole transitions at optical frequencies. *Phys. Rev. Lett.* **114**, 163903 (2015).
2. Dodson, C. et al. Magnetic dipole and electric quadrupole transitions in the trivalent lanthanide series: Calculated emission rates and oscillator strengths. *Phys. Rev. B.* **86**, 125102 (2012).
3. Taminiau, T. et al. Optical antennas direct single-molecule emission. *Nat. Photonics.* **2**, 234-237 (2008).
4. Aigouy, L. et al. Mapping and Quantifying Electric and Magnetic Dipole Luminescence at the Nanoscale. *Phys. Rev. Lett.* **113**, 076101 (2014).
